# Daily alcohol use covaries with daily concentration problems across the lifespan: Findings from the MIDUS Refresher

**DOI:** 10.1093/geroni/igab046.2598

**Published:** 2021-12-17

**Authors:** Jacqueline Mogle, Ashley Linden-Carmichael, Sara Miller, David Almeida

**Affiliations:** 1 Penn State University, University Park, Pennsylvania, United States; 2 Penn State Univerity, State College, Pennsylvania, United States; 3 Pennsylvania State University, University Park, Pennsylvania, United States

## Abstract

Alcohol use is typically associated with impaired cognitive functioning on tasks related to attention and concentration. However, it remains unclear whether these impairments persist across days in ways that are noticeable to the individual. We examined this using the daily diary project of the Midlife in the United States Refresher cohort. Participants (n=710; Mage=50.5; range 25-75) completed 8 nights of telephone-based diaries (Mdiaries=6.87) that included questions about daily alcohol use (“how many drinks did you have today?”) and five items assessing concentration (e.g., “today, did you have difficulty concentrating?”) rated on a scale (1=none of the time to 5=all of the time). Using autoregressive multilevel models, we examined how same and previous day alcohol use related to perceived difficulties with concentration. Greater total alcohol use over the diary period was related to reports of concentration problems (b=.31, SE=.10, p=.002) though current day (b=-.03, SE=.04, p=.49) and previous day alcohol use (b=.05, SE=.04, p=.23) were not. The association between previous day use and concentration problems was qualified by an interaction with total alcohol use (b=-.07, SE=.03, p=.002). Individuals who drank less alcohol in general, experienced greater perceived concentration problems following the days on which they did drink (b=.14, SE=.07, p=.03) relative to those who drank more alcohol across the diary period (b=-.04, SE=.04, p=.36). This relationship did not vary based on age, sex, or education. These results suggest that daily alcohol use could impair concentration across days, particularly for those adults who tend to consume less alcohol.

